# Relationship between the *T_C_* of Smart Meta-Superconductor Bi(Pb)SrCaCuO and Inhomogeneous Phase Content

**DOI:** 10.3390/nano11051061

**Published:** 2021-04-21

**Authors:** Honggang Chen, Mingzhong Wang, Yao Qi, Yongbo Li, Xiaopeng Zhao

**Affiliations:** Smart Materials Laboratory, Department of Applied Physics, Northwestern Polytechnical University, Xi’an 710129, China; 2017100698@mail.nwpu.edu.cn (H.C.); wangmingzhongsuper@163.com (M.W.); qiyao@mail.nwpu.edu.cn (Y.Q.); 2014100616@mail.nwpu.edu.cn (Y.L.)

**Keywords:** smart meta-superconductor B(P)SCCO, inhomogeneous phase content, critical transition temperature, EL energy injection, increase amplitude Δ*T_C_*

## Abstract

A smart meta-superconductor Bi(Pb)SrCaCuO (B(P)SCCO) may increase the critical transition temperature (*T_C_*) of B(P)SCCO by electroluminescence (EL) energy injection of inhomogeneous phases. However, the increase amplitude Δ*T_C_* ($\Delta T_{C}=T_{C}-T_{C,pure}$) of *T_C_* is relatively small. In this study, a smart meta-superconductor B(P)SCCO with different matrix sizes was designed. Three kinds of raw materials with different particle sizes were used, and different series of Y_2_O_3_:Sm^3+^, Y_2_O_3_, Y_2_O_3_:Eu^3+^, and Y_2_O_3_:Eu^3+^+Ag-doped samples and pure B(P)SCCO were prepared. Results indicated that the *T_C_* of the Y_2_O_3_ or Y_2_O_3_:Sm^3+^ non-luminescent dopant doping sample is lower than that of pure B(P)SCCO. However, the *T_C_* of the Y_2_O_3_:Eu^3+^+Ag or Y_2_O_3_:Eu^3+^ luminescent inhomogeneous phase doping sample is higher than that of pure B(P)SCCO. With the decrease of the raw material particle size from 30 to 5 μm, the particle size of the B(P)SCCO superconducting matrix in the prepared samples decreases, and the doping content of the Y_2_O_3_:Eu^3+^+Ag or Y_2_O_3_:Eu^3+^ increases from 0.2% to 0.4%. Meanwhile, the increase of the inhomogeneous phase content enhances the Δ*T_C_*. When the particle size of raw material is 5 μm, the doping concentration of the luminescent inhomogeneous phase can be increased to 0.4%. At this time, the zero-resistance temperature and onset transition temperature of the Y_2_O_3_:Eu^3+^+Ag doped sample are 4 and 6.3 K higher than those of pure B(P)SCCO, respectively.

## 1. Introduction

Since the discovery of superconductivity, raising the critical transition temperature (*T_C_*) of superconductivity to obtain a room-temperature superconductor has been the main goal of superconductivity research. In 2011, Cavalleri et al. used a mid-infrared femtosecond laser pulse to induce the transformation of La_1.675_Eu_0.2_Sr_0.125_CuO_4_ from a non-superconducting phase to a transient superconducting phase [1]. Subsequently, they used similar experimental methods to induce transient superconducting transitions in La_1.84_Sr_0.16_CuO_4_, YBa_2_Cu_3_O_6.5_, and K_3_C_60_ [2,3,4]. Since then, the use of light to change the superconducting properties of materials has been gradually recognized by researchers. In 2015, German researchers observed the superconductivity of 203 K in a sulfur hydride system at 155 Gpa [5], and they obtained a superconductivity of 250 K at 170 GPa for LaH_10_ in 2019 [6]. In 2020, Dias et al. achieved a superconducting transition of 287.7 K at approximately 267 GPa in a carbonaceous sulfur hydride system [7]. Although the *T_C_* continues to refresh, the ultra-high pressure, extremely small sample size, and material instability under ambient pressure limit further applications.

The known stable high-temperature superconductors are all layered copper oxides with a perovskite-like structure. Among them, Bi-based superconductors (BSCCO) with a *T_C_* beyond 100 K have become the most promising material for scientific and industrial applications due to their several advantages, such as high *T_C_*, low oxygen sensitivity, and absence of rare earth [8,9,10]. Bi-based superconductors have three superconducting phases of Bi2201 (*T_C_* = 20 K), Bi2212 (*T_C_* = 85 K), and Bi2223 (*T_C_* = 110 K). They are symbiotic with one another, and a pure single phase is difficult to obtain [11,12,13,14]. By partial substitution of Bi by Pb, the volume fraction of the high-temperature phase Bi2223 can be increased, thus making the Bi2223 phase easier to synthesize and increasing its stability [15,16]. Although Bi-based superconductors are called high-temperature superconductors, a large gap still exists between their *T_C_* and practical applications. Hence, researchers have done substantial work to improve their *T_C_*. At present, the most commonly used method is chemical doping, such as doping Cs [17], Al [18], SnO_2_ [19], ZrO_2_ [20], Ca_2_B_2_O_5_ [21], and others in BSCCO. However, most of the dopants are unstable at high temperatures and will react with superconductors; the results are not ideal.

Metamaterial is a kind of composite material with an artificial structure [22,23,24]. With the development of metamaterials, researchers have found that the concept of metamaterials can be used to design superconductors and a metamaterial superconductor can exhibit a higher *T_C_* [25,26,27,28,29,30]. In 2007, our group doped inorganic electroluminescence (EL) material in a superconductor to increase *T_C_* via EL. Jiang et al. [31] introduced a ZnO EL material into B(P)SCCO superconductors for the first time. The results showed that the *T_C_* of B(P)SCCO was slightly reduced due to the low EL intensity and the high doping concentration of ZnO. In recent years, we constructed a smart meta-superconductor MgB_2_. The conventional superconductor MgB_2_ is doped with Y_2_O_3_:Eu^3+^, Y_2_O_3_:Eu^3+^+Ag sheets, and Y_2_O_3_:Eu^3+^ rods [32,33,34] of different sizes. The results showed that the doping of Y_2_O_3_:Eu^3+^ EL materials increases the *T_C_* of MgB_2_. We believe that the Y_2_O_3_:Eu^3+^ materials generate an EL under the action of an external electric field and that EL energy injection promotes the formation of Cooper pairs; the *T_C_* of MgB_2_ is improved via EL [35,36,37,38,39]. At the same time, a smart meta-superconductor B(P)SCCO consists of the B(P)SCCO matrix and an inhomogeneous phase Y_2_O_3_:Eu^3+^ or Y_2_O_3_:Eu^3+^+Ag was constructed in BSCCO superconductors. It was shown that the addition of Y_2_O_3_:Eu^3+^ and Y_2_O_3_:Eu^3+^+Ag inhomogeneous phase increases the *T_C_* of B(P)SCCO [40]. However, the *T_C_* of the pure B(P)SCCO prepared is low, the increase amplitude Δ*T_C_* ($\Delta T_{C}=T_{C}-T_{C,pure}$) is small, and the influencing factors are not clear.

On the basis of previous research, a smart meta-superconductor consists of an inhomogeneous phase and the B(P)SCCO matrix with different sizes is designed in this study. Three kinds of raw materials with different particle sizes are used, and three different series of samples are prepared by solid-state sintering. The transition width and *T_C_* of the prepared samples are consistent with the literature [41,42,43]. The *T_C_* of Y_2_O_3_:Sm^3+^ and Y_2_O_3_ non-luminescent dopant doping sample is lower than that of pure B(P)SCCO ($\Delta T_{C}<0$). However, the *T_C_* of Y_2_O_3_:Eu^3+^ and Y_2_O_3_:Eu^3+^+Ag luminescent inhomogeneous phases doping sample is higher than that of pure B(P)SCCO ($\Delta T_{C}>0$). With the decrease of the raw material particle size, the particle size of the B(P)SCCO superconducting matrix in the prepared samples decreases, and the doping content of the Y_2_O_3_:Eu^3+^+Ag and Y_2_O_3_:Eu^3+^ inhomogeneous phases can increase. Meanwhile, the increase of luminescent inhomogeneous phases content amplifies the increase amplitude Δ*T_C_* of *T_C_*.

## 2. Model

**Figure 1:**
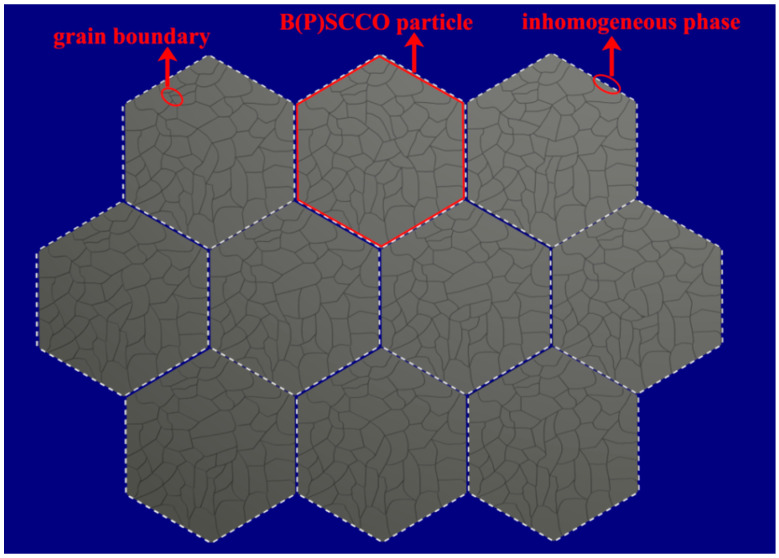
The model of the smart meta-superconductor B(P)SCCO.

On the basis of the idea of metamaterials, a smart meta-superconductor B(P)SCCO model is constructed, as shown in Figure 1. The model is composed of B(P)SCCO superconducting particles of the matrix material and a Y_2_O_3_:Eu^3+^+Ag or Y_2_O_3_:Eu^3+^ luminescent inhomogeneous phase. The gray hexagons in the figure represent B(P)SCCO superconducting particles. The superconducting particles are composed of numerous small crystal grains, and the black lines in the gray hexagons represent the B(P)SCCO grain boundaries. The luminescent inhomogeneous phase is located around the B(P)SCCO superconducting particles and is represented by a discontinuous white color. When measuring the *R-T* curve of the samples, under the action of an external electric field, the B(P)SCCO superconducting particles act as microelectrodes, which excite the EL of the luminescent inhomogeneous phase. Moreover, the formation of electron pairs can be promoted via EL energy injection, and the *T_C_* of B(P)SCCO will be improved. By adjusting the external electric field to control the EL and adjust the *T_C_*, a smart meta-superconductor is realized.

By changing the size of the matrix material in the smart meta-superconductor B(P)SCCO model, more luminescent inhomogeneous phases can be accommodated in the same space volume as the size of the B(P)SCCO superconducting particle decreases. In addition, the distribution of the inhomogeneous phases is more uniform. Hence, the formation of electron pairs is enhanced, and the increase amplitude Δ*T_C_* of *T_C_* will be further increased.

## 3. Experiment

### 3.1. Preparation of Luminescent Inhomogeneous Phases and Non-Luminescent Dopants

The preparation process and related characterization of the Y_2_O_3_:Eu^3+^+Ag topological luminophore inhomogeneous phase were described in [33,40]. The same preparation process is used by changing the raw materials to obtain Y_2_O_3_:Eu^3+^ luminescent inhomogeneous phase and Y_2_O_3_ and Y_2_O_3_:Sm^3+^ non-luminescent dopants.

### 3.2. Preparation of Pure B(P)SCCO Superconductors

A certain amount of raw materials according to the molar ratio Bi_2_O_3_:PbO:SrCO_3_:CaCO_3_:CuO = 0.85:0.4:2.00:2.00:3.00 was weighed and placed in an agate tank for 500 r/min ball milling for 20, 50, or 80 h. The raw material of the 20-h ball milling was dried and sieved with a 500-mesh stainless steel sieve to obtain B(P)SCCO raw material S1 with a particle size of approximately 30 μm. The raw material after 50-h ball milling was filtered and dried to obtain B(P)SCCO raw material S2 with a particle size of approximately 15 μm. Meanwhile, the raw material of 80-h ball milling was filtered and dried to obtain B(P)SCCO raw material S3 with a particle size of approximately 5 μm. Then, raw materials S1, S2, and S3 were kept at 840 °C for 50 + 50 h, respectively. They were fully ground once in the middle to obtain three kinds of B(P)SCCO calcined powder. The calcined powder was sufficiently ground and pressed into a pellet of 12 mm diameter and 2 mm thickness. The pellet was kept at 840 °C in the air for 30 + 120 h. It was milled and pressed again in the middle to obtain a pure B(P)SCCO sample.

### 3.3. Preparation of Doping B(P)SCCO Superconducting Samples

A certain amount of (0.0012, 0.0018, or 0.0024 g) Y_2_O_3_:Eu^3+^+Ag, Y_2_O_3_:Eu^3+^ luminescent inhomogeneous phases or Y_2_O_3_, Y_2_O_3_:Sm^3+^ dopants was fully mixed with three kinds of B(P)SCCO calcined powder (0.6 g). Then, the mixture was pressed into a pellet. The pellet was kept at 840 °C in the air for 30 + 120 h. It was milled and pressed again in the middle to obtain samples doped with different dopants.

### 3.4. Characterization

X-ray diffraction (XRD) patterns within the range of 3° ≤ 2θ ≤ 60° were obtained using an Hitachi XRD-7000 diffractometer with Cu Kα radiation at a scanning rate of 0.1 °/s. A FEI Verios G4 scanning electron microscope (SEM) was used to analyze the microstructure of the sintered samples. Resistivity versus temperature measurements were performed on each of the samples using the standard four-probe technique in a liquid helium cryogenic system. In addition, 0.1 mA current was applied. Keithley digital nanovoltmeter was used to measure the high resolution voltage across the sample.

In the experiment, the particle size of the raw materials S1, S2, and S3 obtained by ball milling and drying decreased in turn. The samples prepared with raw materials S1, S2, and S3 were recorded as A series samples (as shown in Table 1), B series samples (as shown in Table 2), and C series samples (as shown in Table 3), respectively.

## 4. Results and Discussion

**Figure 2:**
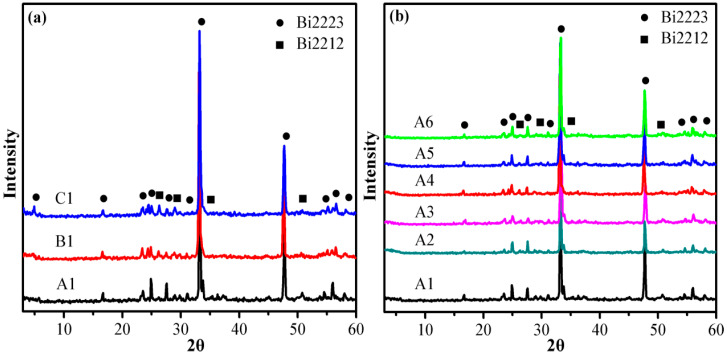
XRD patterns of samples. (**a**) XRD patterns of pure B(P)SCCO A1, B1, and C1 prepared by the raw materials S1, S2, and S3. (**b**) XRD patterns of pure B(P)SCCO (A1) and B(P)SCCO doped with 0.2 wt% Y_2_O_3_:Sm^3+^ (A2), 0.2 wt% Y_2_O_3_ (A3), 0.2 wt% Y_2_O_3_:Eu^3+^ (A4), 0.2 wt% Y_2_O_3_:Eu^3+^+Ag (A5), and 0.3 wt% Y_2_O_3_:Eu^3+^+Ag (A6).

Figure 2a shows the XRD patterns of pure B(P)SCCO A1, B1, and C1 prepared by the solid-state sintering of raw materials S1, S2, and S3. The peak intensities and positions of the diffraction pattern demonstrate that the main phase of prepared pure B(P)SCCO is the high-temperature phase Bi2223 (the volume fraction is approximately 95%). A small amount of the low-temperature phase Bi2212 is detected, and no other impurity phases exist. Moreover, the particle size of the raw material does not affect the phase formation of prepared samples. The high-temperature phase Bi2223 and the low-temperature phase Bi2212 are marked with a circle and a square, respectively. The calculation formula for the relative volume fraction of the high-temperature phase Bi2223 and the low-temperature phase Bi2212 of all samples is as follows [44,45]:

$$\mathrm{Bi}2223\left(\%\right)\approx \frac{\sum I\left(Bi2223\right)}{\sum I\left(Bi2223\right)+\sum I\left(Bi2212\right)}\times 100\%,$$

$$\mathrm{Bi}2212\left(\%\right)\approx \frac{\sum I\left(Bi2212\right)}{\sum I\left(Bi2223\right)+\sum I\left(Bi2212\right)}\times 100\%,$$

where *I* is the intensity of the Bi2223 phase and the Bi2212 phase in the XRD pattern. Figure 2b depicts the XRD patterns of doped samples (A1, A2, A3, A4, A5, and A6) prepared by raw material S1. The addition of the Y_2_O_3_:Sm^3+^ and Y_2_O_3_ non-luminescent dopants or the Y_2_O_3_:Eu^3+^ and Y_2_O_3_:Eu^3+^+Ag luminescent inhomogeneous phases does not introduce other impurity phases.

**Figure 3:**
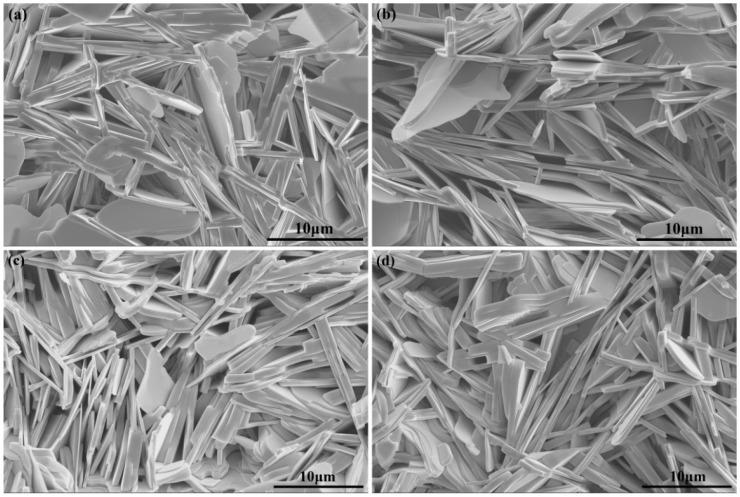
SEM images of samples. (**a**–**c**) SEM images of pure B(P)SCCO A1, B1, and C1 prepared from the raw materials S1, S2, and S3. (**d**) SEM image of B(P)SCCO doped with 0.4 wt% Y_2_O_3_:Eu^3+^+Ag (C7) prepared from the raw material S3.

The microstructure is one of the important properties of high-temperature superconductors. Figure 3a–c demonstrate the SEM images of pure B(P)SCCO A1, B1, and C1 prepared from raw materials S1, S2, and S3, respectively. In all samples, the dominant structures are plate-like structures randomly distributed due to the presence of the pores. As the size of the raw materials S1, S2, and S3 decreases, the size of the plate-like structures in the prepared sample diminishes. Meanwhile, the pores increase, thus being able to accommodate more Y_2_O_3_:Eu^3+^+Ag and Y_2_O_3_:Eu^3+^ inhomogeneous phases in the same space volume. In addition, the inhomogeneous phase distribution around the B(P)SCCO superconducting matrix is more uniform. Figure 3d shows the SEM image of B(P)SCCO doped with 0.4 wt% Y_2_O_3_:Eu^3+^+Ag (C7) prepared from the raw material S3. The doping of Y_2_O_3_:Eu^3+^+Ag inhomogeneous phase does not affect the microstructure of B(P)SCCO.

**Figure 4:**
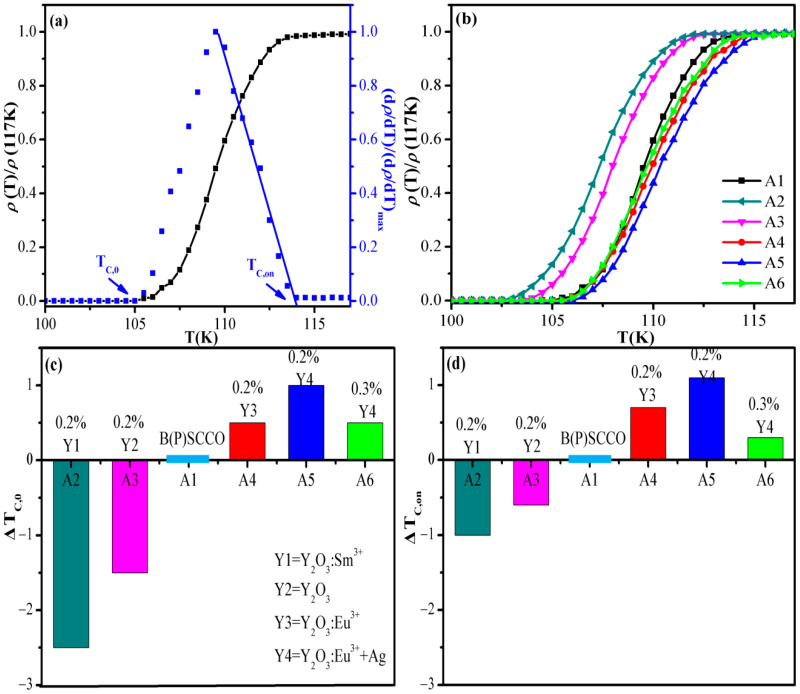
Superconductivity of A series samples. (**a**) Temperature dependence of normalized *dρ*/*dT* of pure B(P)SCCO (A1). (**b**) Temperature-dependent normalized resistivity curves of pure B(P)SCCO (A1) and B(P)SCCO doped with 0.2 wt% Y_2_O_3_:Sm^3+^ (A2), 0.2 wt% Y_2_O_3_ (A3), 0.2 wt% Y_2_O_3_:Eu^3+^ (A4), 0.2 wt% Y_2_O_3_:Eu^3+^+Ag (A5), and 0.3 wt% Y_2_O_3_:Eu^3+^+Ag (A6). (**c**,**d**) The Δ*T_C,_*_0_ and Δ*T_C,on_* of A2, A3, A4, A5, and A6 relative to A1.

The standard four-probe method is used to test the *R-T* curve of the prepared samples from 50 K to room temperature. In the normal state region, all samples show the linear temperature dependence characteristic of Cu-oxide-based high-temperature superconductors and a superconducting transition between 90–115 K. Figure 4a shows the normalized *R-T* curve (the black curve) of the pure B(P)SCCO (A1) prepared from raw material S1. Through the relationship between temperature and normalized *dρ*/*dT* (the blue curve in Figure 4a), zero-resistance temperature *T_C,_*_0_ and onset transition temperature *T_C,on_* can be obtained. Zero-resistance temperature *T_C,_*_0_ is the temperature at which the resistance has just completely dropped to zero during the cooling process. Meanwhile, onset transition temperature *T_C,on_* is the temperature at which the *R–T* curve deviates from linear behavior. The *T_C,_*_0_ and *T_C,on_* of the pure B(P)SCCO (A1) are 105 and 113.7 K, respectively, and the transition width is 8.7 K, which is consistent with the literature [41,42,43]. Figure 4b presents the normalized *R-T* curve of pure B(P)SCCO (A1) and B(P)SCCO doped with 0.2 wt% Y_2_O_3_:Sm^3+^ (A2), Y_2_O_3_ (A3), Y_2_O_3_:Eu^3+^ (A4), Y_2_O_3_:Eu^3+^+Ag (A5), and 0.3 wt% Y_2_O_3_:Eu^3+^+Ag (A6). Figure 4c,d present the Δ*T_C,_*_0_ and Δ*T_C,on_* of A2, A3, A4, A5, and A6 relative to the pure B(P)SCCO (A1), respectively. The specific values of the *T_C_* are shown in Table 1. From the chart, we can observe that the Y_2_O_3_:Sm^3+^ and Y_2_O_3_ non-luminescent dopants doping make the *T_C_* of B(P)SCCO lower (Δ*T_C_* < 0). However, the addition of the Y_2_O_3_:Eu^3+^+Ag and Y_2_O_3_:Eu^3+^ inhomogeneous phases increases *T_C_* (Δ*T_C_* > 0), which may be due to the luminescent inhomogeneous phase distributed around the B(P)SCCO superconducting particles to form a metamaterial structure with a special response. When measuring the *R-T* curve of the sample, B(P)SCCO superconducting particles act as the microelectrode, which excites the EL of the luminescent inhomogeneous phase. In addition, the formation of electron pairs is promoted via EL energy injection so that the *T_C_* of B(P)SCCO is improved. The *T_C,_*_0_ and *T_C,on_* of 0.2 wt% Y_2_O_3_:Eu^3+^+Ag-doped sample (A5) are higher than 0.3% Y_2_O_3_:Eu^3+^+Ag-doped sample (A6). All the *R-T* curves display characteristic behavior of a percolative transition [46,47,48]; the present experimental results can be explained by observing a broad percolative transition [49,50]. In the percolative transition, the *T_C,on_* is related to some regions where the *T_C_* is higher than the average *T_C_*, and these regions match the *T_C_* of Bi2223. The *T_C,_*_0_ is related to the percolation, which occurs at a lower temperature. The underlying mechanism of the experimental results is not particularly clear and requires further exploration in subsequent research.

**Figure 5:**
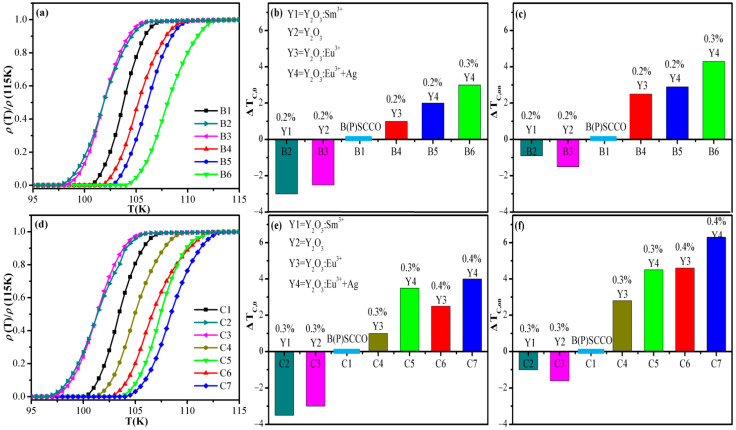
Superconductivity of B and C series samples. (**a**) Normalized *R-T* curves of pure B(P)SCCO (B1) and B(P)SCCO doped with 0.2 wt% Y_2_O_3_:Sm^3+^ (B2), 0.2 wt% Y_2_O_3_ (B3), 0.2 wt% Y_2_O_3_:Eu^3+^ (B4), 0.2 wt% Y_2_O_3_:Eu^3+^+Ag (B5), and 0.3 wt% Y_2_O_3_:Eu^3+^+Ag (B6). (**b**,**c**) The Δ*T_C,_*_0_ and Δ*T_C,on_* of B2, B3, B4, B5, and B6 relative to B1. (**d**) Normalized *R-T* curves of pure B(P)SCCO (C1) and B(P)SCCO doped with 0.3 wt% Y_2_O_3_:Sm^3+^ (C2), 0.3 wt% Y_2_O_3_ (C3), 0.3 wt% Y_2_O_3_:Eu^3+^ (C4), 0.3 wt% Y_2_O_3_:Eu^3+^+Ag (C5), 0.4 wt% Y_2_O_3_:Eu^3+^ (C6), and 0.4 wt% Y_2_O_3_:Eu^3+^+Ag (C7). (**e**,**f**) The Δ*T_C,_*_0_ and Δ*T_C,on_* of C2, C3, C4, C5, C6, and C7 relative to C1.

Figure 5a demonstrates the normalized *R-T* curve of pure B(P)SCCO (B1) and 0.2 wt% Y_2_O_3_:Sm^3+^ (B2), Y_2_O_3_ (B3), Y_2_O_3_:Eu^3+^ (B4), Y_2_O_3_:Eu^3+^+Ag (B5), and 0.3 wt% Y_2_O_3_:Eu^3+^+Ag (B6) doped samples. Figure 5b,c represents increase amplitudes Δ*T_C,_*_0_ and Δ*T_C,on_* of B2, B3, B4, B5, and B6 relative to the pure B(P)SCCO (B1), respectively. Figure 5d shows the normalized *R-T* curve of pure B(P)SCCO (C1) and B(P)SCCO doped with 0.3 wt% Y_2_O_3_:Sm^3+^ (C2), 0.3 wt% Y_2_O_3_ (C3), 0.3 wt% Y_2_O_3_:Eu^3+^ (C4), 0.3 wt% Y_2_O_3_:Eu^3+^+Ag (C5), 0.4 wt% Y_2_O_3_:Eu^3+^ (C6), and 0.4 wt% Y_2_O_3_:Eu^3+^+Ag (C7). Figure 5e,f represents increase amplitudes Δ*T_C,_*_0_ and Δ*T_C,on_* of C2, C3, C4, C5, C6, and C7 relative to the pure B(P)SCCO (C1), respectively. The *T_C_* of each sample is shown in Table 2 and Table 3. The non-luminescent dopants Y_2_O_3_ and Y_2_O_3_:Sm^3+^ doping make the *T_C,_*_0_ and *T_C,on_* of B(P)SCCO decrease by 2–4 and 1–2 K, respectively. And as the doping content of the non-luminescent dopants increases, the *T_C_* of B(P)SCCO decreases more. While the Y_2_O_3_:Eu^3+^ and Y_2_O_3_:Eu^3+^+Ag luminescent inhomogeneous phase doped samples have a higher *T_C,_*_0_ and *T_C,on_* than the pure sample. *T_C,on_* increases more. The *T_C_* of Y_2_O_3_:Eu^3+^+Ag doping is higher than that of Y_2_O_3_:Eu^3+^ doping. In addition, within a certain content range, the Δ*T_C,_*_0_ and Δ*T_C,on_* increases as the content of the luminescent inhomogeneous phase increases.

The particle size of raw material S1 is 30 μm, and the optimal doping concentration of the inhomogeneous phase in the sample prepared by raw material S1 is 0.2%. At this time, compared with pure B(P)SCCO (A1), the *T_C,_*_0_ and *T_C,on_* of B(P)SCCO doped with the Y_2_O_3_:Eu^3+^ inhomogeneous phase increase by 0.5 and 0.7 K, respectively. Moreover, the Y_2_O_3_:Eu^3+^+Ag inhomogeneous phase doped samples increase by 1 and 1.1 K, respectively. However, the particle size of raw material S3 is 5 μm, and the doping concentration of the inhomogeneous phase in the sample prepared by raw material S3 can be increased to 0.4%. At this time, compared with pure B(P)SCCO (C1), the *T_C,_*_0_ and *T_C,on_* of B(P)SCCO doped with the Y_2_O_3_:Eu^3+^ inhomogeneous phase increase by 2.5 and 4.6 K, and the Y_2_O_3_:Eu^3+^+Ag inhomogeneous phase doped samples increase by 4 and 6.3 K, respectively. As the particle size of raw material decreases from 30 to 5 μm, the particle size of the B(P)SCCO superconducting matrix in the prepared sample gradually decreases, and the inhomogeneous phase doping concentration increases from 0.2% to 0.4%. At the same time, the increase of the inhomogeneous phase concentration enhances the Δ*T_C_*.

## 5. Conclusions

We designed a smart meta-superconductor B(P)SCCO with different matrix sizes. Three different series of samples were prepared by solid-state sintering using three kinds of particle size raw materials. The main phase of the prepared samples is the high-temperature phase Bi2223 and contains a small amount of the low-temperature phase Bi2212. In addition, the microstructure consists of randomly distributed EL inhomogeneous phase and a plate-like B(P)SCCO matrix. *R-T* tests found that the *T_C_* of Y_2_O_3_:Eu^3+^+Ag or Y_2_O_3_:Eu^3+^ luminescent inhomogeneous phase doping sample is higher than that of pure B(P)SCCO. Meanwhile, the *T_C_* of the Y_2_O_3_ or Y_2_O_3_:Sm^3+^ non-luminescent dopant doping sample is lower than that of pure B(P)SCCO. As the particle size of raw material decreases from 30 to 5 μm, the particle size of the B(P)SCCO superconducting matrix decreases gradually, and the doping content of the Y_2_O_3_:Eu^3+^+Ag and Y_2_O_3_:Eu^3+^ inhomogeneous phases increases from 0.2% to 0.4%. Meanwhile, the growth of the inhomogeneous phase content further enhances the increase amplitude Δ*T_C_*. When the raw material particle size is 5 μm, the inhomogeneous phase added to the prepared sample can be increased to 0.4%. At this time, the *T_C,_*_0_ and *T_C,on_* of Y_2_O_3_:Eu^3+^+Ag doped sample are 4 and 6.3 K higher than those of pure B(P)SCCO, respectively.

## Figures and Tables

**Table 1:** Dopant, doping concentration, *T_C,_*_0_, and *T_C,on_* of A series samples prepared from raw material S1 (30 μm).

Sample	Dopant	Doping Concentration (wt%)	*T_C,_*_0_/K	*T_C,on_*/K	Δ*T_C,_*_0_/K	Δ*T_C,on_*/K
**A1**	None	0	105	113.7	0	0
**A2**	Y_2_O_3_:Sm^3+^	0.2	102.5	112.7	−2.5	−1
**A3**	Y_2_O_3_	0.2	103.5	113.1	−1.5	−0.6
**A4**	Y_2_O_3_:Eu^3+^	0.2	105.5	114.4	0.5	0.7
**A5**	Y_2_O_3_:Eu^3+^+Ag	0.2	106	114.8	1	1.1
**A6**	Y_2_O_3_:Eu^3+^+Ag	0.3	105.5	114	0.5	0.3

**Table 2:** Dopant, doping concentration, *T_C,_*_0_, and *T_C,on_* of B series samples prepared from raw material S2 (15 μm).

Sample	Dopant	Doping Concentration (wt%)	*T_C,_*_0_/K	*T_C,on_*/K	Δ*T_C,_*_0_/K	Δ*T_C,on_*/K
**B1**	None	0	100.5	107.9	0	0
**B2**	Y_2_O_3_:Sm^3+^	0.2	97.5	107	−3	−0.9
**B3**	Y_2_O_3_	0.2	98	106.4	−2.5	−1.5
**B4**	Y_2_O_3_:Eu^3+^	0.2	101.5	110.4	1	2.5
**B5**	Y_2_O_3_:Eu^3+^+Ag	0.2	102.5	110.8	2	2.9
**B6**	Y_2_O_3_:Eu^3+^+Ag	0.3	103.5	112.2	3	4.3

**Table 3:** Dopant, doping concentration, *T_C,_*_0_, and *T_C,on_* of C series samples prepared from raw material S3 (5 μm).

Sample	Dopant	Doping Concentration (wt%)	*T_C,_*_0_/K	*T_C,on_*/K	Δ*T_C,_*_0_/K	Δ*T_C,on_*/K
**C1**	None	0	100	107.6	0	0
**C2**	Y_2_O_3_:Sm^3+^	0.3	96.5	106.6	−3.5	−1
**C3**	Y_2_O_3_	0.3	97	106	−3	−1.6
**C4**	Y_2_O_3_:Eu^3+^	0.3	101	110.4	1	2.8
**C5**	Y_2_O_3_:Eu^3+^+Ag	0.3	103.5	112.1	3.5	4.5
**C6**	Y_2_O_3_:Eu^3+^	0.4	102.5	112.2	2.5	4.6
**C7**	Y_2_O_3_:Eu^3+^+Ag	0.4	104	113.9	4	6.3

## Data Availability

The date presented in this study are available on reasonable request from the corresponding author.
